# GAUGE-Annotated Microbial Transcriptomic Data Facilitate Parallel Mining and High-Throughput Reanalysis To Form Data-Driven Hypotheses

**DOI:** 10.1128/mSystems.01305-20

**Published:** 2021-03-23

**Authors:** Zhongyou Li, Katja Koeppen, Victoria I. Holden, Samuel L. Neff, Liviu Cengher, Elora G. Demers, Dallas L. Mould, Bruce A. Stanton, Thomas H. Hampton

**Affiliations:** a Department of Microbiology and Immunology, Geisel School of Medicine at Dartmouth, Hanover, New Hampshire, USA; Duke University

**Keywords:** *Pseudomonas aeruginosa*, biofilms, bioinformatics, gene expression, genomics

## Abstract

The NCBI Gene Expression Omnibus (GEO) provides tools to query and download transcriptomic data. However, less than 4% of microbial experiments include the sample group annotations required to assess differential gene expression for high-throughput reanalysis, and data deposited after 2014 universally lack these annotations. Our algorithm GAUGE (general annotation using text/data group ensembles) automatically annotates GEO microbial data sets, including microarray and RNA sequencing studies, increasing the percentage of data sets amenable to analysis from 4% to 33%. Eighty-nine percent of GAUGE-annotated studies matched group assignments generated by human curators. To demonstrate how GAUGE annotation can lead to scientific insight, we created GAPE (GAUGE-annotated Pseudomonas aeruginosa and Escherichia coli transcriptomic compendia for reanalysis), a Shiny Web interface to analyze 73 GAUGE-annotated P. aeruginosa studies, three times more than previously available. GAPE analysis revealed that *PA3923*, a gene of unknown function, was frequently differentially expressed in more than 50% of studies and significantly coregulated with genes involved in biofilm formation. Follow-up wet-bench experiments demonstrate that *PA3923* mutants are indeed defective in biofilm formation, consistent with predictions facilitated by GAUGE and GAPE. We anticipate that GAUGE and GAPE, which we have made freely available, will make publicly available microbial transcriptomic data easier to reuse and lead to new data-driven hypotheses.

**IMPORTANCE** GEO archives transcriptomic data from over 5,800 microbial experiments and allows researchers to answer questions not directly addressed in published papers. However, less than 4% of the microbial data sets include the sample group annotations required for high-throughput reanalysis. This limitation blocks a considerable amount of microbial transcriptomic data from being reused easily. Here, we demonstrate that the GAUGE algorithm could make 33% of microbial data accessible to parallel mining and reanalysis. GAUGE annotations increase statistical power and, thereby, make consistent patterns of differential gene expression easier to identify. In addition, we developed GAPE (GAUGE-annotated Pseudomonas aeruginosa and Escherichia coli transcriptomic compendia for reanalysis), a Shiny Web interface that performs parallel analyses on P. aeruginosa and E. coli compendia. Source code for GAUGE and GAPE is freely available and can be repurposed to create compendia for other bacterial species.

**Author Video**: An author video summary of this article is available.

## INTRODUCTION

The NCBI Gene Expression Omnibus (GEO) is a public archive of high-throughput functional genomics data that includes microarray and RNA sequencing (RNA-seq) data submitted by the research community (1, 2). To date, GEO stores data from more than 130,000 studies, with over 3.8 million samples. Each submitted study has a unique GEO series (GSE) record that provides minimum information as defined in the Minimum Information About a Microarray Experiment (MIAME) (3) and Minimum Information About a Next-generation Sequencing Experiment (MINSEQE) (https://www.ncbi.nlm.nih.gov/geo/info/MIAME.html) guidelines. Unfortunately, these guidelines do not require researchers to identify experimental groups explicitly, impeding our ability to use computer programs to reanalyze these data. Recognizing this deficiency and others, the GEO staff has manually added this information to selected GEO records, turning them into curated GEO “DataSets” (GDS) and “Profiles,” which allow advanced data display and analysis provided on the GEO Web portal. In our previous work, we launched the user-friendly Shiny Web application ScanGEO, which permits investigators to interrogate the differential expression of a custom list of genes across all GDS of a species or a subset of studies of interest (4). ScanGEO dramatically facilitates the rapid analysis of a relatively large number of data sets, which increases statistical power and allows for the identification of differential gene expression patterns not possible when examining smaller individual data sets.

**FIG 1:**
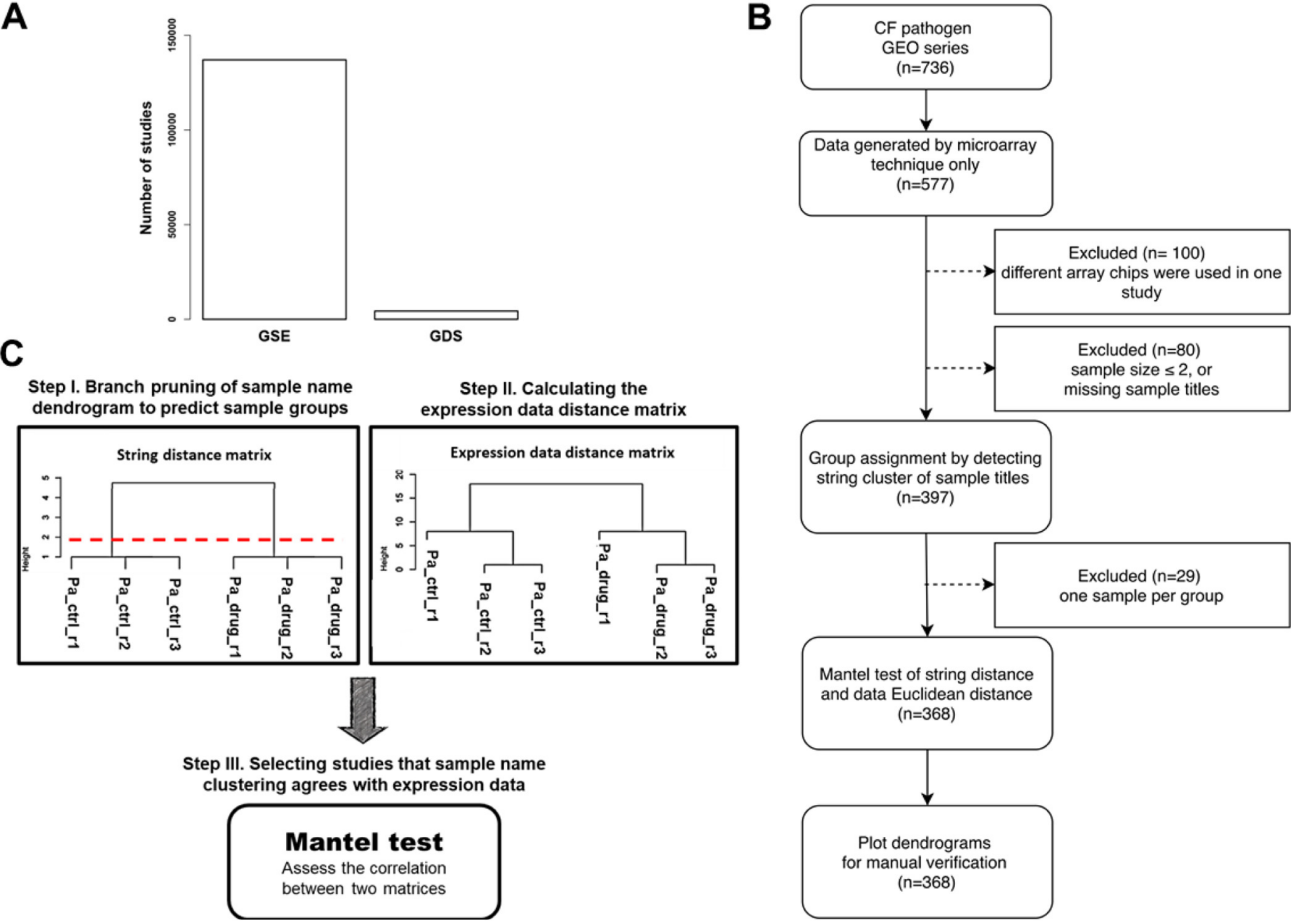
GAUGE is designed to increase the number of curated data by detecting the sample groups of annotatable studies. (A) There are disproportionate numbers of all GEO series (GSE) records and those manually curated with sample group information (GDS). (B) Schematic overview of the microarray data selection strategy and manual verification of the algorithm (see Materials and Methods for further details). (C) The three core steps of GAUGE generate a text string distance matrix for sample groups using text-mining techniques, calculate a gene expression distance matrix using experimental data, and use the Mantel test to assess the correlation between the two matrices to determine whether a study is auto-annotatable.

While the GDS records are excellent resources for investigators to form data-driven hypotheses (1), less than 4% of all GSE records have been manually curated into GEO DataSets (Fig. 1A). Perhaps because manual curation is a labor-intensive and time-consuming process, data curation ceased in 2015, despite the rapidly growing number of submitted studies. Thus, there is an urgent need to develop an automatic way to select and annotate studies suitable for high-throughput reanalysis. Based on our primary research interest, we chose to focus our analysis on three common pathogens that increase morbidity and mortality in cystic fibrosis (CF) patients: Pseudomonas aeruginosa, Staphylococcus aureus, and Candida albicans (5). Besides infecting CF patients, these opportunistic pathogens infect other immunocompromised individuals, such as patients with chronic obstructive pulmonary disease (COPD) or burn wounds (6–8).

Others have created tools that facilitate manual curation or automatically identify a text pattern associated with a particular kind of sample metadata. Zhao Li et al. developed a Web-based manual curation platform to coordinate curation efforts from different curators to reduce curation time (9). A GEO Web portal analysis tool, GEO2R, requires a user to assign sample groups manually and identifies differentially expressed genes one study at a time; however, GEO2R supports only microarray studies (1). Another microarray-only tool, GEOracle, provides a semimanual process for users to inspect and modify the predicted annotation (10). Pattern-matching techniques have previously been used to extract sample attributes, such as tissue, gender, and age, from the metadata of human samples. However, this process requires the manual identification of domain-specific vocabularies (11). Taken together, none of the existing solutions provide an automated, high-throughput, cross-species, and cross-platform approach to annotate GEO Series records. Accordingly, we have developed an algorithm, GAUGE (general annotation using text/data group ensembles) that automatically annotates GEO microbial data sets, including microarray and RNA sequencing studies, thereby increasing the percentage of archived data sets amenable to analysis from 4% to 33%.

In this study, we report the development of GAUGE, which focuses on automated sample group detection rather than the curation or extraction of sample information. We hypothesized that the sample titles from the same sample group are similar to each other and different from the sample titles of other groups. We also hypothesized that correctly clustered sample titles should parallel the clustering of expression data from the same experiment. Using publicly available microbial microarray and RNA-seq studies, we demonstrated the ability of GAUGE to identify annotatable data and assign the sample groups with high accuracy.

Existing algorithms to cluster sample replicates and extract labels (10, 11) use text pattern-matching techniques to identify a reference group for comparison. Since reference naming conventions are complex and domain dependent, text-matching approaches, therefore, require the investigator to manually define and update the vocabulary that will be used, limiting the scope of applicable studies. For example, GEOracle (10) excludes studies whose samples cannot be classified as control group versus experimental group. To the best of our knowledge, there is no algorithm like GAUGE, which uses sample titles and expression data to cluster samples and is therefore fully automatically applicable to data in any domain.

The sample group information was further used to perform an analysis of variance (ANOVA) on each GAUGE-annotated study, which allows the user to identify differential gene expression patterns. Using the GAUGE-annotated ANOVA compendium, we identified underappreciated P. aeruginosa genes that are frequently differentially expressed with large fold changes (FCs). More intriguingly, correlation analysis and Kyoto Encyclopedia of Genes and Genomes (KEGG) (12) pathway enrichment analysis of the compendium revealed a functional prediction for *PA3923*, a P. aeruginosa gene encoding a hypothetical protein. We conducted experiments that supported the prediction that PA3923 is a novel protein involved in P. aeruginosa biofilm formation. The GAUGE-annotated P. aeruginosa ANOVA compendium created in this study makes three times more P. aeruginosa data accessible to parallel mining and reanalysis. GAUGE annotations increase statistical power and, thereby, make consistent patterns of differential gene expression easier to identify. The GAUGE algorithm can also be used to create high-throughput reanalysis compendia for other organisms and platforms.

## RESULTS

### GAUGE accurately selects and annotates sample groups, extending the number of studies that can be systematically analyzed.

**FIG 2:**
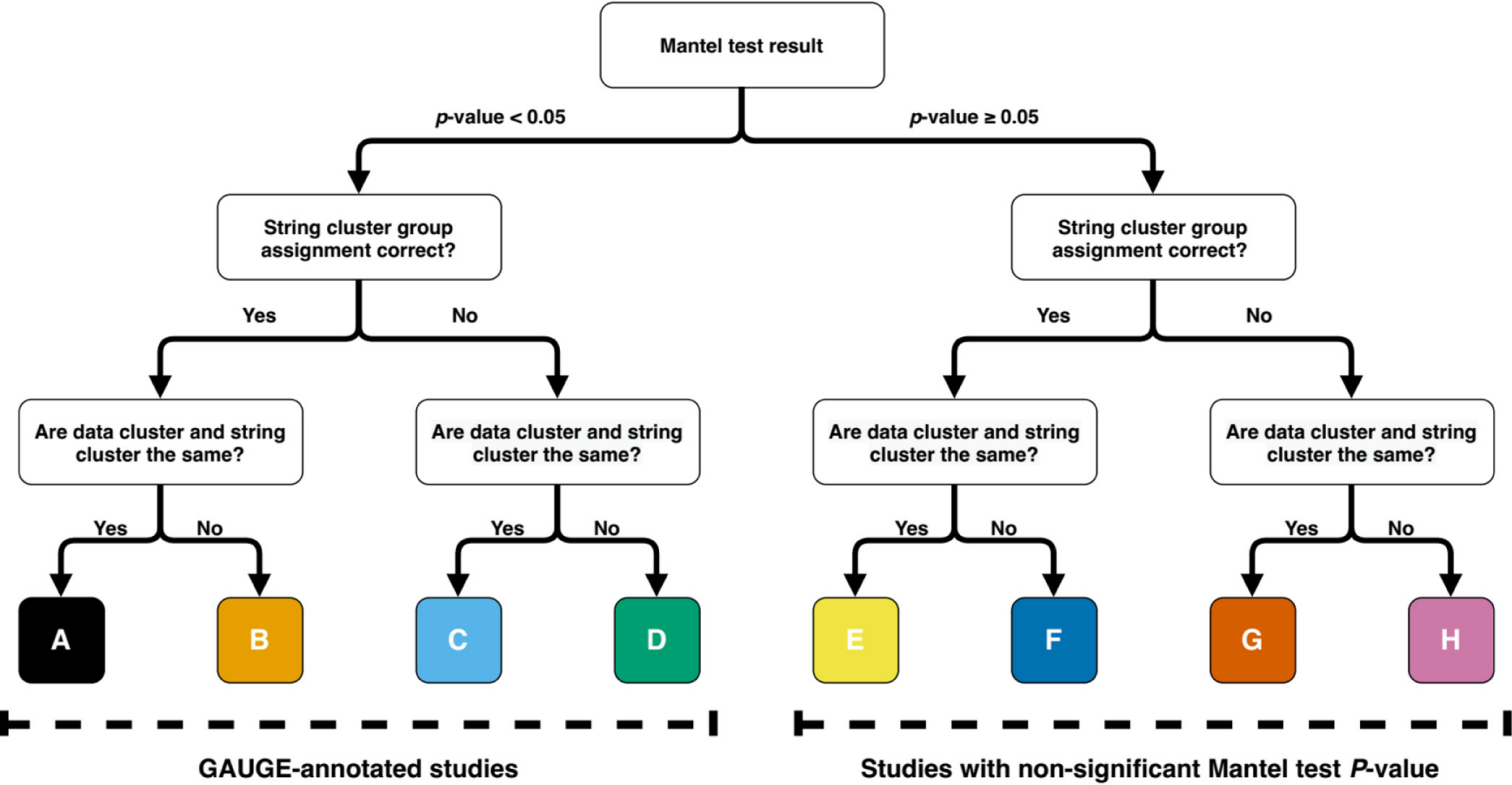
Decision tree for manual verification. For each tested study, dendrograms of string distance and Euclidean distance are generated and subjected to manual verification according to the decision tree, with eight possible decisions. The conventional significance level of 0.05 is used for the Mantel test to define GAUGE-annotated studies. The eight possible decisions’ color theme is applied to Fig. 3 and 4.

**FIG 3:**
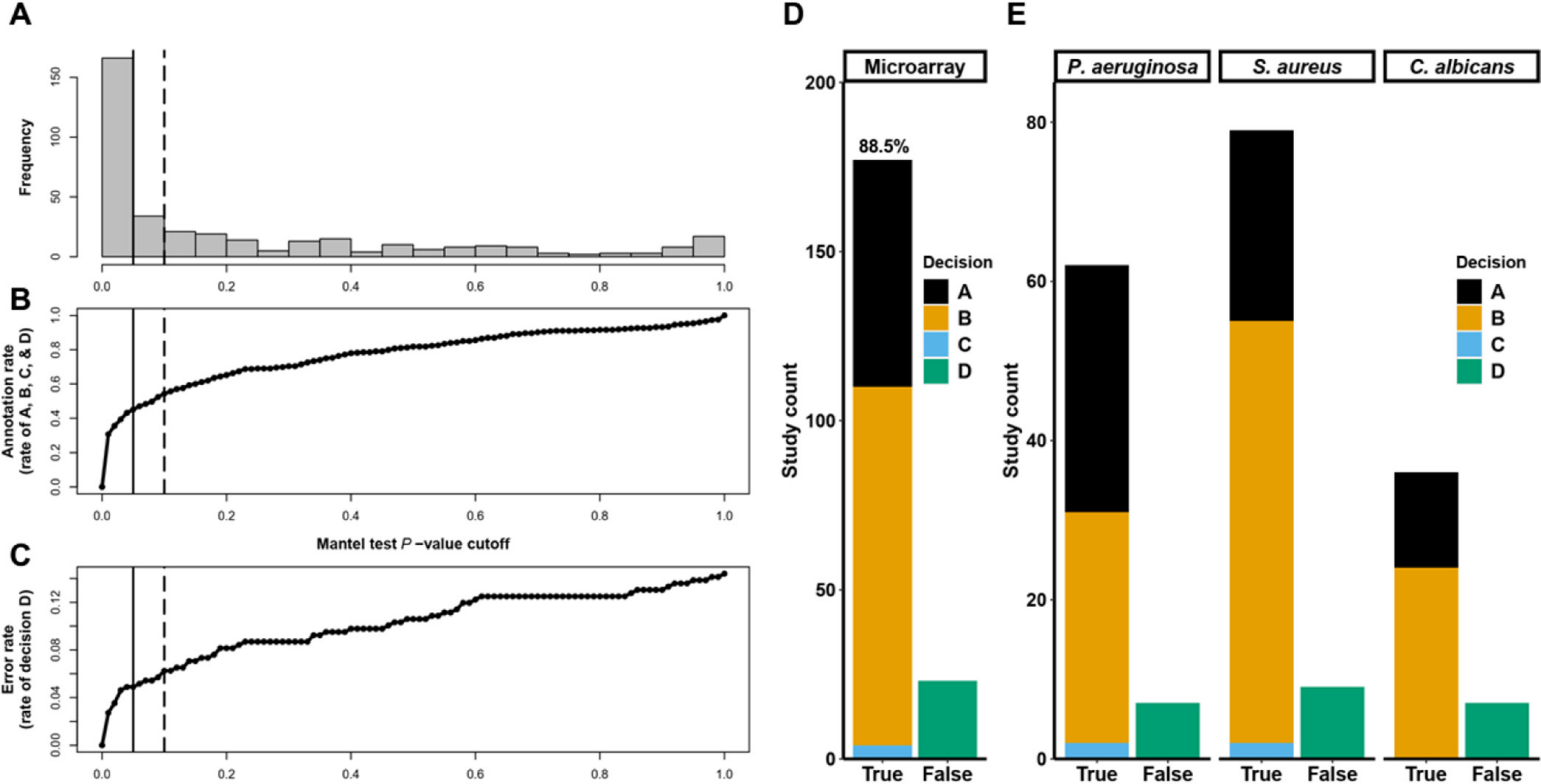
GAUGE selects microarray studies and detects sample groups with high accuracy. (A) *P* value distribution of 368 Mantel tests of microarray studies. (B and C) Annotation rate (B) and error rate (C) of the manual verification result of 368 microarray studies as the discrimination Mantel test *P* value threshold increases from 0 to 1 in 0.01 increments. The annotation rate (B) and error rate (C) are the rates of decisions A, B, C, and D or decision D only, respectively. The vertical solid and dashed lines indicate the significance levels of 0.05 and 0.1, respectively. (D) Summary of the manual verification result of GAUGE-annotated microarray studies with a Mantel test *P* value smaller than 0.1. (E) Results broken down by species. The verification decisions, shown in different colors, fall into true or false annotation groups. The corresponding precision level of GAUGE-annotated studies, 88.5%, is labeled.

To establish GAUGE, we used microarray studies because they have a standardized matrix format containing expression data. We downloaded 577 microarray studies from three common CF pathogens and removed those that did not meet our selection criteria (Fig. 1B; see Materials and Methods for further details). For each of the 368 remaining studies, we used GAUGE to generate sample group predictions and calculate Mantel test *P* values. The Mantel test assesses the correlation between the string distance matrix of sample titles and the Euclidean distance matrix of gene expression data (Fig. 1C). The string distance matrix quantifies the similarity between sample titles, and the Euclidean distance matrix quantifies the similarity between gene expression profiles of samples. We hypothesized that a substantial correlation between these two independent distance matrices was unlikely to occur by chance and that a significant positive correlation provides evidence that sample title clustering might be capable of discerning sample groups. For studies with small Mantel test *P* values, the sample group assignments are most likely meaningful, and the gene expression data reflect the experimental designs. The conventional *P* value cutoff, smaller than 0.05, was used to define significance, as shown in the decision tree used for manual verification (Fig. 2). In our 368 Mantel test analyses, the *P* value distribution was skewed toward 0 (Fig. 3A), suggesting that our algorithm systematically outperforms chance.

To examine GAUGE performance under different alpha levels of the Mantel test, human curators independently compared the dendrograms of string distance and Euclidean distance to score the accuracy of automatic sample group assignments using the defined decision tree with eight decisions: A through H (Fig. 2). Studies with a Mantel test *P* value smaller than alpha level fell into categories A, B, C, or D and were considered GAUGE-annotated studies. With this verification data, we observed that increasing the Mantel test *P* value cutoff from 0.05 to 0.1 improved the GAUGE auto-annotation rate by 9% (Fig. 3B) (from 45% to 54%), while increasing the error rate, the rate of decision D, by only 1% (Fig. 3C) (from 5% to 6%). Furthermore, with a significance level of 0.1, we achieved a precision rate of 88.5% (Fig. 3D). The precision rate was defined as the cumulative percentage of decisions A, B, and C in GAUGE-annotated studies. The curated outcome distribution patterns were similar across three different CF pathogens (Fig. 3E).

To achieve a high validation accuracy, each study was assigned to at least two curators, and their curation results were compared and integrated to find the final decisions. To validate 368 studies, seven curators spent more than 10 h in total. In contrast, GAUGE finished the task in 5 min with a single R session and a single core running on a Linux machine.

### GAUGE annotates RNA sequencing studies with similar levels of performance.

Next, we tested the ability of GAUGE to annotate RNA sequencing data, and we estimated the performance. To skip fastq file download and read alignment, we retrieved expression data for all available microbial RNA-seq studies from refine.bio (13; https://www.refine.bio). The RNA-seq data archived in refine.bio were uniformly processed into gene-level count tables similar to standard series matrix files of microarray studies. We downloaded all 302 available microbial RNA-seq studies and applied the same criteria used for microarray studies (Fig. 1B) to select those suitable for GAUGE analysis. A total of 139 microbial RNA-seq studies, including experiments with Saccharomyces cerevisiae, P. aeruginosa, and Escherichia coli, were used for GAUGE annotation and manual verification.

**FIG 4:**
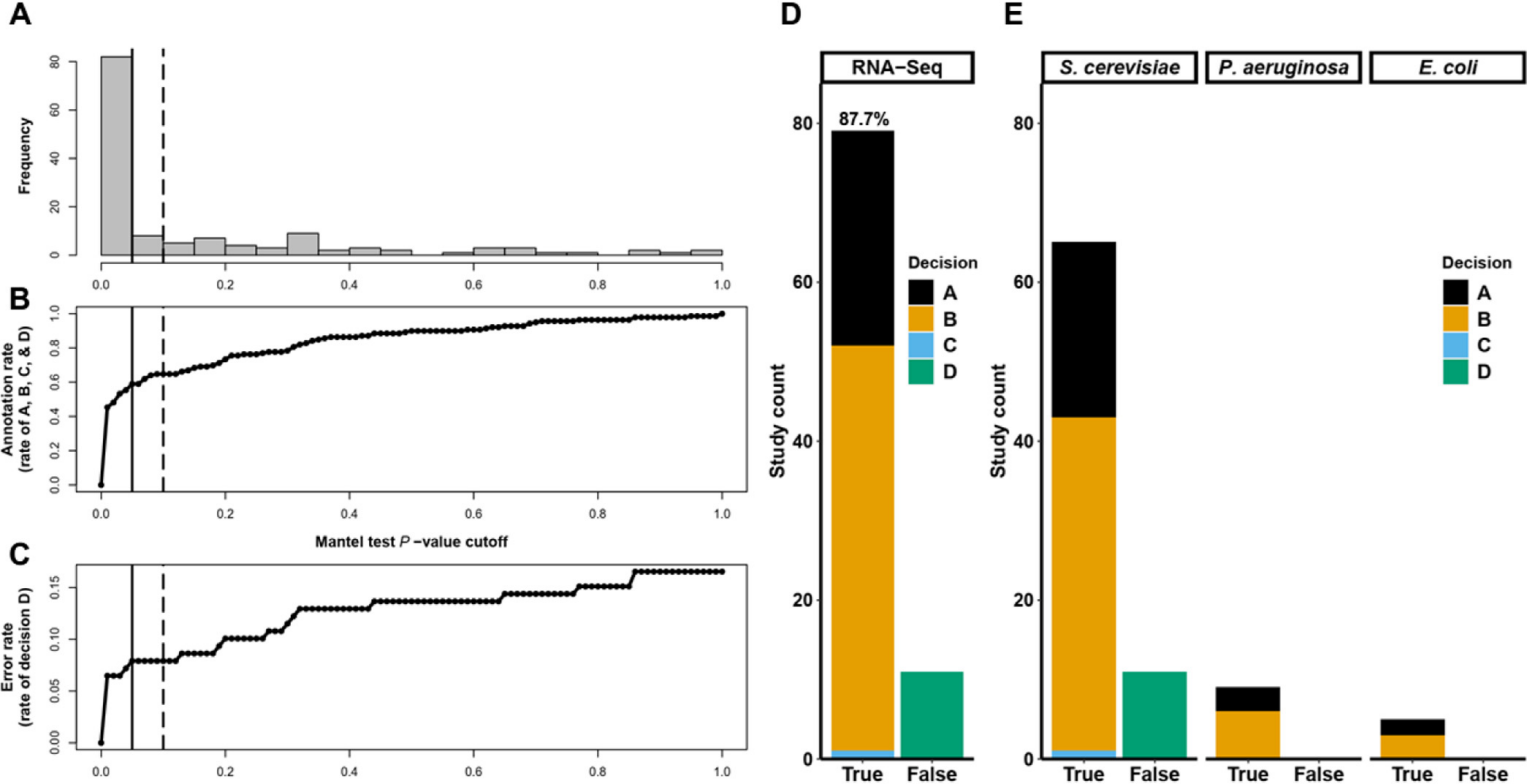
GAUGE automatically selects and detects sample groups from RNA-seq studies with levels of performance similar to those of microarray studies. (A) *P* value distribution of 139 Mantel tests of RNA-seq studies. (B and C) Annotation rate (B) and error rate (C) of the manual verification result of 139 RNA-seq studies as the discrimination Mantel test *P* value threshold increases from 0 to 1 in 0.01 increments. The annotation rate (B) and error rate (C) are the rates of decisions A, B, C, and D and decision D only, respectively. The vertical solid and dashed lines indicate the significance levels of 0.05 and 0.1, respectively. (D) Summary of the manual verification result of GAUGE-annotated RNA-seq studies with a Mantel test *P* value smaller than 0.1. (E) Results broken down by species. The verification decisions, shown in different colors, fall into true or false annotation groups. The corresponding precision level of GAUGE-annotated studies, 87.7%, is labeled.

Applying GAUGE to RNA-seq studies from refine.bio, we observed a distribution of Mantel test *P* values similar to that used for microarray studies (Fig. 4A). With a Mantel test significance level of 0.1, GAUGE automatically annotated 90 out of 139 studies (65%) (Fig. 4B), with an error rate (decision D) of only 8% (Fig. 4C). Increasing the *P* value cutoff from 0.05 to 0.1 raised the annotation rate by 6% without increasing the error rate (Fig. 4C). Among GAUGE-annotated studies, we achieved an 87.7% precision level (Fig. 4D), with an overall and species-level outcome distribution pattern of manual verification (Fig. 4E) similar to the one observed for microarray studies.

Taken together, GAUGE automatically selected and annotated 33% of microbial studies, including microarray and RNA sequencing data sets, tested in this report. Among them, 89% of GAUGE annotations matched group assignments generated by human curators. This significantly increases the number of studies amenable to systematic analysis and our ability to generate biological insights, as evidenced below.

### GAUGE-annotated ANOVA compendium reveals frequently differentially expressed genes with undefined functions.

We used the sample group information detected by GAUGE, with a *P* value cutoff of 0.1, to perform ANOVA. The ANOVA identified differentially expressed genes in a GAUGE-annotated P. aeruginosa compendium of 73 studies, including microarray and RNA-seq studies, for a total of 1,003 samples. The maximum absolute log_2_ fold change (log_2_ FC) between any two groups was calculated for each gene in every study. The top 10 most frequently differentially expressed genes (Table 1) were found to be differentially expressed in more than half of the studies in the compendium, with at least a median fold change of 3.7 (log_2_ FC = 1.9). Intriguingly, genes that are frequently differentially expressed often have no known functional annotation in the Pseudomonas genome database (14). For example, in the top 10 most frequently differentially expressed genes, two genes encode proteins with probable functions, predicted by sequence similarity, and one hypothetical gene, *PA3923*, has no known function. These genes respond to numerous treatment conditions and are therefore likely to play important roles in P. aeruginosa biology. Notably, *PA3923* had a median fold change of >7, and the increase in *PA3923* was associated with an increase in the expression of other genes in P. aeruginosa that play a role in biofilm formation.

**TABLE 1:** Top 10 most frequently differentially expressed genes in 73 GAUGE-annotated P. aeruginosa studies

Standing	Locus tag	Gene product description	No. of studies^*a*^	Median log_2_ FC^*b*^
1	PA0105	Cytochrome *c* oxidase, subunit II	43	2.882226
2	PA1041	Probable outer membrane protein precursor^*c*^	41	2.765857
3	PA3723	Probable FMN oxidoreductase^*c*^	41	2.654894
4	PA2851	Translation elongation factor P	41	1.929715
5	PA2445	Glycine cleavage system protein P2	40	3.101212
6	PA4296	Two-component response regulator, PprB	40	3.019888
7	PA3418	Leucine dehydrogenase	40	2.789049
8	PA5172	Ornithine carbamoyltransferase, catabolic	40	2.625181
9	PA1984	NAD^+^-dependent aldehyde dehydrogenase	39	3.385404
10	PA3923	Hypothetical protein	39	2.942898

a Differentially expressed genes are determined with an FDR of <0.05 using an ANOVA with sample groups detected by the auto-annotation algorithm.

b Median log_2_ fold change of gene expression in those differentially expressed studies. The log_2_ fold change was defined as the maximum absolute log_2_ fold change between any two groups for each gene in every study.

c The gene annotation is from the Pseudomonas Genome Database. A function is proposed based on the conserved amino acid motif, structural feature, or limited sequence similarity to an experimentally studied gene. FMN, flavin mononucleotide.

### Correlation and pathway enrichment analysis suggest that hypothetical protein PA3923 plays a role in biofilm formation, a clinically relevant phenotype.

P. aeruginosa biofilms play important roles in clinical outcomes of patients with COPD, cystic fibrosis, chronic wounds, or catheter-associated infection (7, 8, 15). Biofilm formation contributes to bacterial persistence, and antibiotic resistance determinants make P. aeruginosa recalcitrant to the host immune system and antibiotic treatment (7). Thus, there is a critical need to identify new target genes in P. aeruginosa that facilitate opportunistic infection and biofilm formation.

**FIG 5:**
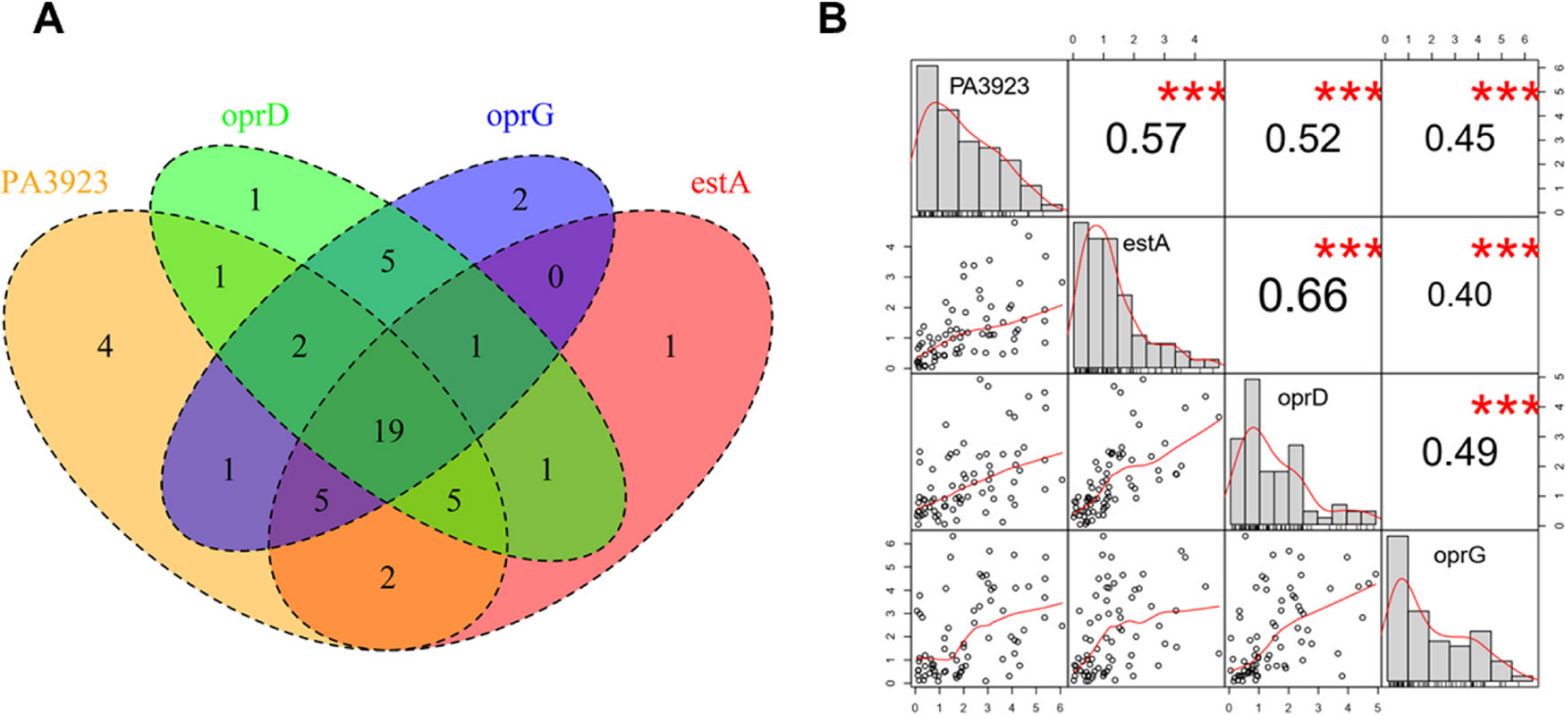
The P. aeruginosa compendium reveals the correlation between laminin receptors. (A) Venn diagram showing the number of studies in the P. aeruginosa compendium that have a differentially expressed *PA3923*, *oprD*, *oprG*, or *estA* signal. (B) Correlation matrix of log_2_ fold changes of the four genes across all studies in the compendium. The numbers in the upper triangle are the Pearson correlation coefficients of gene pairs. ***, *P* value < 0.001.

Several lines of evidence suggest that PA3923 may be involved in biofilm formation. First, a proteomic investigation has shown that PA3923 protein is exclusively detected in P. aeruginosa grown in biofilm cultures but not in planktonic cells (16). Second, a recent report using murine laminin as a bait reveals that three well-characterized proteins (EstA, OprD, and OprG) in the P. aeruginosa outer membrane, as well as PA3923, have high binding affinities for laminin (17). Laminin is a major component of the host extracellular matrix (ECM), and bacterial adherence to certain host ECM molecules plays an important role in biofilm formation (18). Third, our GAUGE-annotated P. aeruginosa ANOVA compendium revealed that in 49% of studies (19 out of 39) in which *PA3923* was differentially expressed, *estA*, *oprD*, and *oprG* were also differentially expressed (Fig. 5A). A Pearson correlation analysis of the log_2_ fold change of the transcripts of four laminin-binding proteins across all studies in the compendium shows that the expression of the four genes are highly correlated with each other (*P* < 0.001) (Fig. 5B). Even though *estA*, *oprD*, and *oprG* are not included in the KEGG biofilm formation pathway (12), *estA* and *oprD* mutants have biofilm formation defects compared to wild-type P. aeruginosa (19, 20). Moreover, the protein level of *oprG* is upregulated in P. aeruginosa biofilms compared to that in planktonic cells (21). KEGG pathway enrichment analysis using Fisher’s exact test across the GAUGE-annotated P. aeruginosa ANOVA compendium shows that the biofilm formation pathway is enriched in differentially expressed genes (false-discovery rate [FDR] < 0.05) in 18 studies. *PA3923* was differentially expressed in 11 of these 18 studies (61%) (Table 2). This suggests that studies in which the biofilm formation pathway is enriched have a disproportionately higher rate of differentially expressed *PA3923* than the rate of the whole compendium (39/73, 53%). Interestingly, for four of the top seven studies in which our analysis revealed an enriched P. aeruginosa biofilm formation pathway signal, a connection to biofilm formation was not mentioned in the original publications (Table 2). Taken together, our analysis suggests that genes producing laminin-binding proteins at the P. aeruginosa surface, such as EstA, OprD, OprG, and PA3923, are highly coregulated and may play roles in biofilm formation. Thus, our analyses support the hypothesis that *PA3923*, a gene of unknown function, encodes a laminin-binding protein and, therefore, promotes biofilm formation.

**TABLE 2:** GAPE identifies studies in which the biofilm formation pathway is enriched

GSE no.	Title^*a*^	*PA3923* DE^*b*^	Biofilm formation pathway reported^*c*^	Reference
GSE21966	Transcriptional profiling of P. aeruginosa isolated from 3 individuals with cystic fibrosis over time		✓	32
GSE62970	Expression data of clinical P. aeruginosa isolates grown *in vitro* in minimal glucose medium	✓		NA
GSE67006	Expression data from P. aeruginosa wild type and Δ*anr* grown as biofilms on ΔF508 cystic fibrosis bronchial epithelial cells (CFBEs)		✓	33
GSE28719	Gene expression of P. aeruginosa Δ*PA1006*/*nbvF* mutant in the absence and presence of nitrate	✓	✓	34
GSE39044	Regulon of transcriptional regulator PA2449 in P. aeruginosa PAO1	✓		35
GSE8408	Transcriptomic analysis of the sulfate starvation response in P. aeruginosa	✓		36
GSE78255	Gene expression data from P. aeruginosa PAO1 and mutator (Δ*mutS*) evolved for 940 generations in LB with and without subinhibitory concentrations of ciprofloxacin (0.05 μg/ml)	✓		37

a The top 7 out of 18 studies have an enriched P. aeruginosa biofilm formation pathway signal. The enrichments are determined with a Fisher’s exact test FDR of <0.05, and studies are ranked from the smallest FDR.

b Checked if the hypothetical gene *PA3923* is differentially expressed (DE) in the study.

c Checked if the study has been reported with an enriched biofilm formation pathway signal in publications.

### Experimental evidence suggests that PA3923 mediates biofilm on laminin-coated surfaces.

**FIG 6:**
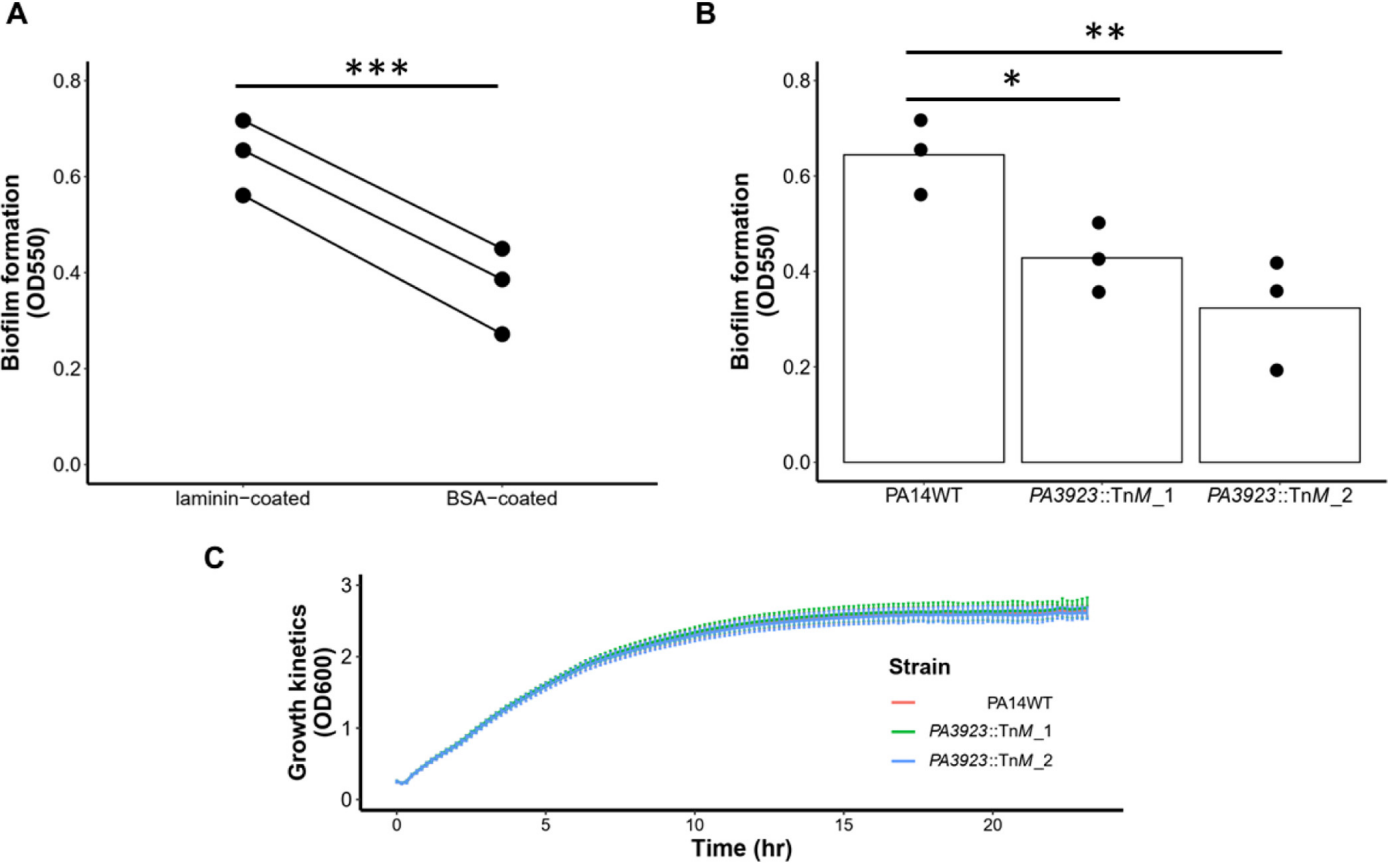
*PA3923* transposon insertion mutants have biofilm formation defects on a laminin-coated surface. (A) Quantification of biofilm formation by the wild-type PA14 strain on laminin-coated ELISA plates and BSA-coated ELISA plates. Each data point indicates the average from six technical replicates. Lines connect data points from the same clone, a biological replicate, of the wild-type PA14 strain under each condition. (B) Biofilm formation by the PA14 wild type (PA14WT) and two *PA3923* transposon insertion mutants (*PA3923*::Tn*M*_1 and *PA3923*::Tn*M*_2) on laminin-coated ELISA plates. Each dot represents a unique clone from a single colony of each strain. Biofilms were quantified using the crystal violet 96-well plate biofilm assay (OD_550_). (C) Growth kinetics (OD_600_) of bacterial strains used in this study. Three single colonies of each strain were picked randomly to generate three clones as three biological replicates for all experiments. Bars and lines in panels B and C represent the averages from three biological replicates, with six technical replicates each. Error bars indicate standard deviations (SD). Linear models in R were used to calculate *P* values. *, *P < *0.05; **, *P < *0.01; ***, *P < *0.001.

To test the hypothesis that laminin promotes P. aeruginosa biofilm formation, we performed crystal violet (CV) biofilm assays on laminin-coated 96-well enzyme-linked immunosorbent assay (ELISA) plates or on bovine serum albumin (BSA)-coated 96-well ELISA plates as a control (22). Biofilm formation was significantly more robust on laminin-coated wells than on BSA-coated wells (Fig. 6A). To test the hypothesis that PA3923 protein facilitates the formation of biofilms on laminin-coated surfaces, we used two *PA3923* transposon insertion mutants from the P. aeruginosa PA14 transposon library (PA14NR set) (23) and compared the abilities of wild-type PA14 and the two mutants to form biofilms on laminin-coated wells. The mutants, *PA3923*::Tn*M*_1 and *PA3923*::Tn*M*_2, have insertion sites at the 5′ end and the 3′ end of their *PA3923* genes, respectively (23). Biofilms formed on laminin by both mutants were significantly smaller (by 33.5% and 49.8%, respectively) than those formed by the wild-type PA14 strain (Fig. 6B). The mutants’ reduction in biofilm formation could not be attributed to a growth defect of planktonic bacteria (Fig. 6C). These experiments confirm the hypothesis that PA3923 enhances biofilm formation on laminin-coated surfaces, as predicted by our analysis of the GAUGE-annotated P. aeruginosa ANOVA compendium.

### GAUGE-annotated ANOVA compendia and the GAUGE source code are freely available.

To facilitate the reanalysis of publicly available data for novel hypothesis generation by microbiologists, we provide the GAUGE-annotated P. aeruginosa and E. coli ANOVA compendia generated in this study. E. coli studies measured on two Affymetrix platforms, GEO accession no. GPL3154 and GPL199, were downloaded and annotated since they contain the largest number of experiment series. The compendia and the source code can be accessed at https://github.com/DartmouthStantonLab/GAUGE/. The GAUGE source code can easily be repurposed to annotate other microbial species, including other clinically relevant ESKAPE pathogens, such as Enterococcus faecium, S. aureus, Klebsiella pneumoniae, Acinetobacter baumannii, and *Enterobacter* spp.

### GAPE, a Shiny app to explore the GAUGE-annotated ANOVA compendia, is freely available.

The Shiny Web tool used in this analysis can be accessed at https://iamsoshiny.shinyapps.io/gape/. This free tool is particularly useful for users without R programming skills to navigate the GAUGE-annotated P. aeruginosa and E. coli ANOVA compendia and to find differential gene expression as well as pathway enrichment signals of interests. The source code for GAPE is freely available at https://github.com/DartmouthStantonLab/GAPE/.

## DISCUSSION

The algorithm, data compendia, and online analysis tool presented in this report contribute to the goal of advancing the FAIR (findability, accessibility, interoperability, and reusability) data paradigm (24) in three ways. First, the GAUGE algorithm is a general-purpose tool, can be applied to any annotation problem where textual metadata are expected to correlate with quantitative observational data, and is applicable to both prokaryotic and eukaryotic species. Second, compendia for P. aeruginosa and E. coli can be downloaded and explored in a full-featured statistical programming environment such as R. Finally, our Web portal, GAPE, further extends FAIR data goals because it requires no special skills to use. The strengths, limitations, and future development of each of these systems are further discussed below.

The main strength of GAUGE is that it correctly annotates a substantial number of unannotated studies without time-consuming human intervention. In our proof-of-principle experiment, GAUGE speedily annotated about 33% of microbial transcriptomic studies, with an error rate of less than 7%. As of this writing, the GEO database contains over 130,000 data series (GSE), including 1,200 studies of E. coli. Assuming that it takes about 15 min to manually curate a given study into the GDS format, a human might assign sample group information to about 30 studies in an 8-h workday. Annotating the E. coli studies would therefore take more than 40 days, which would make it very difficult for anyone to undertake a systematic assessment of E. coli transcriptomic responses. In contrast, GAUGE can automatically annotate all suitable E. coli studies and result in a GAUGE-annotated compendium with a total of 400 data sets in 15 min of CPU time.

The primary limitation of GAUGE is that it can annotate studies only with informative sample names. For example, GAUGE cannot detect sample groups in studies with random strings or sample titles that are all numeric. In addition, GAUGE can annotate studies only in which gene expression responses correlate with sample groups. Therefore, GAUGE requires higher levels of replication and consistent transcriptional responses as experimental designs become increasingly complex.

By design, GAUGE is agnostic about what sample groups mean. For example, groups that contain words like “reference” or “treatment” are not treated in any special way. This allows GAUGE to identify significant differences between groups in any study, including those with no obvious references or many possible references. This flexibility is extremely important in the context of data reuse, where investigators may be interested in group comparisons that were not of interest to the investigators that performed the experiments and named the samples. Notably, tools like GAPE use the GAUGE algorithm to rapidly identify a small number of studies with differential gene expression. Once identified, these studies can be analyzed with tools like GEO2R (1), GEOquery (25), and limma (26), which require the user to identify a specific reference group, a step that may require reading the experimental methods published with the data.

Intriguingly, we found that the studies with incorrect auto-annotations usually had complex experimental designs with many sample groups. Generally speaking, GAUGE annotations that were in error were similar to correct annotations but failed to resolve all experimental groups. In any case, a systematic analysis of a large compendium is mostly insensitive to a small number of annotation errors. For example, in our proof of principle, we were successful in making correct predictions about the function of PA3923 despite an estimated error rate of about 7%.

GAUGE can effectively annotate publicly available transcriptomic data sets, as evidenced by our analysis of the P. aeruginosa compendium. Our GAUGE-annotated ANOVA compendia are interoperable with sophisticated platforms like the R statistical programming language, which has a significant learning curve. To make our compendia useful to a broader audience, we have built online tools that easily perform differential gene expression and pathway enrichment analyses. Also, we provide the R source code of GAUGE to enable users to create compendia for other species.

Here, we have demonstrated that GAUGE provides automated and reliable detection of sample groups with unprecedented performance and speed. We expect that over 33% of GEO studies (39,000) can be auto-annotated by GAUGE, a huge improvement over the current situation in which fewer than 4% have group annotation. Increasing the pool of annotated studies greatly extends our ability to study patterns of differential gene expression.

We have shown that systematic analysis, facilitated by GAUGE, can identify genes that may have been overlooked in the literature but that nonetheless may play important biological roles. For example, GAUGE revealed that *PA3923*, a gene of unknown function, was differentially expressed in more than 50% of studies and significantly coregulated with genes involved in biofilm formation. Our follow-up wet-bench experiments demonstrate that *PA3923* does in fact play a role in biofilm formation. However, each of the other genes in Table 1 requires additional scrutiny. Genes that are more likely to respond in common experimental designs are more likely to shed light on questions under study than those that do not. In the same way, though we have demonstrated GAUGE in the context of P. aeruginosa and E. coli, we see no reason why one could not apply GAUGE to S. cerevisiae, which has over 68,000 samples in GEO, or to other clinically relevant species. Thus, we anticipate that GAUGE and GAPE, which we have made freely available, will make publicly available microbial transcriptomic data easier to reuse and lead to new data-driven hypotheses.

## MATERIALS AND METHODS

The core concept of GAUGE is shown in Fig. 1C. All analyses were conducted in the R software environment, except for Entrez Direct (Edirect) (27). Edirect provides access to the NCBI database from a UNIX terminal window, allowing the user to parse and download information from the GEO database. The R packages and parameters that we used for developing the algorithm are described below.

### Data collection and selection.

We used Edirect (27) to parse and collect the metadata, including GSE number, submission date, sample organism, and URL, allowing the download of series matrix files for all existing records. This metadata allowed us to identify 736 studies conducted on three pathogens, P. aeruginosa, S. aureus, and C. albicans. The R package GEOquery was used to retrieve series matrix files (25). At first, we focused on microarray data sets. The series matrix files of array studies were fed into the pipeline shown in Fig. 1B to be filtered. Studies using multiple platforms, e.g., different array chips, in a single experiment were excluded from subsequent analyses. Next, the gene expression data and sample titles of each study were extracted. Studies with low sample sizes or missing series matrix files were also excluded.

All 302 available microbial RNA-seq studies on refine.bio were downloaded. The data were processed by refine.bio with the following steps before download: (i) aggregating the data by experiment and (ii) skipping the transformation and quantile normalization. We selected 139 studies (9 for E. coli, 19 for P. aeruginosa, and 111 for S. cerevisiae) suitable for downstream analysis based on the selection criteria in Fig. 1B.

### GAUGE implementation.

The three core steps are illustrated in Fig. 1C. For each qualifying study, string distance matrices of extracted sample titles were calculated using the stringdist package (28). Sample title clusters were detected by feeding the string distance matrix into the cutreeDynamic algorithm (29), with the argument deepSplit equal to 4 to detect the smallest clusters. Sample title clusters were deemed putative sample groups. Studies with a single sample per group were removed from the analysis since they were not statistically comparable.

Microarray expression values were extracted from downloaded GEO data and log_2_ transformed if not already in log space. For RNA-seq data, we used the edgeR pipeline (30) to perform filtering and library normalization to generate matrices of log_2_-transformed counts per million (CPM). Next, a Euclidean distance matrix of preprocessed gene expression data was generated for each study and compared with the previously calculated string distance matrix using a two-tailed Mantel test with 999 permutations.

GAUGE uses Mantel tests in R (31) to identify cases where sample title clustering and expression data are more correlated than expected by chance. The Mantel test’s null hypothesis is that there is no linear correlation between two matrices. In this case, a small Mantel test *P* value indicates that sample groups identified by stringdist are more similar to groupings of sample data than can be explained by chance, suggesting that group assignment inferred by text mining is correct.

*P* values from Mantel tests and corresponding GSE series numbers were exported as tables for manual verification as defined below. The GAUGE implementation scripts for microarray and RNA-seq experiments can be found at https://github.com/DartmouthStantonLab/GAUGE.

### Manual verification.

For each study, dendrograms of string distance and Euclidean distance were generated. Using these dendrograms and the Mantel test *P* values, human curators manually verified the sample group assignments produced by the algorithm. Curators followed the decision tree in Fig. 2, in which the conventional significance level of 0.05 was chosen to make decisions, A to H. Each study was curated by at least two independent curators to seek consensus. A third curator was involved when there was no consensus.

### Analysis of manual verification result.

The manual verification decisions fell into two categories, GAUGE annotated or not, as shown in Fig. 2, depending on the significance level. We simulated different verification outcomes, as the significance level varies from 0 to 1 in 0.01 increments, and calculated the error rate and annotation rate. The error rate and the annotation rate are the rate of decision D and the rate of decisions A, B, C, and D of the verification result, respectively. The precision rate is the percentage of GAUGE-annotated studies with a significance level of <0.1 that were verified as decisions A, B, and C.

### Building GAUGE-annotated ANOVA compendia.

P. aeruginosa studies with Mantel test *P* values of less than 0.1 were categorized as significantly correlated and correctly annotated. Of these, six studies without proper gene identifiers in the gene expression data table were excluded. Sample group information detected by GAUGE was used to perform an ANOVA for detecting differentially expressed genes in the remaining 64 microarray (Affymetrix platform GPL84) and 9 RNA-seq studies. E. coli studies measured on two Affymetrix platforms, GPL3154 and GPL199, were downloaded and GAUGE annotated since they contain the most abundant experiment series, and ANOVA analysis was performed to generate the compendium.

### GAUGE and Shiny app availability.

The source code of GAUGE and the P. aeruginosa and E. coli ANOVA compendia created in this study can be found at https://github.com/DartmouthStantonLab/GAUGE. GAPE, the Shiny app, deployed on a free server, is accessible at https://iamsoshiny.shinyapps.io/gape. The app can be downloaded from https://github.com/DartmouthStantonLab/GAUGE and run on a local machine. All source code and compendia are available under a GNU general public license (GPLv3).

### Bacterial strains and growth conditions.

The P. aeruginosa strains used in this study are from the nonredundant P. aeruginosa PA14 transposon library (PA14NR set) (23) and were kindly provided by George A. O’Toole. The P. aeruginosa PA14 wild-type strain was steaked onto lysogeny broth (LB) agar plates to obtain single colonies. LB agar plates with 25 μg/ml gentamicin were used to generate single colonies for the two *PA3923* transposon insertion mutants, *PA3923*::Tn*M*_1 and *PA3923*::Tn*M*_2, with mutant IDs 41985 and 53704, respectively, in the PA14NR set. All transposon insertion mutant clones were verified with PCR and Sanger sequencing. Three single colonies of each strain were picked randomly to generate frozen glycerol stocks for three biological replicates. P. aeruginosa strains from frozen stocks were used to inoculate overnight LB liquid cultures at 37°C with shaking at 225 rpm. Gentamicin (20 μg/ml) was added to the culture media for transposon insertion mutants.

### CV biofilm assay.

The biofilm formation abilities of P. aeruginosa strains were assessed using the 96-well plate biofilm assay, as previously described (22). In brief, P. aeruginosa overnight LB cultures were centrifuged, and the cell pellets were washed and resuspended in fresh LB. The optical density at 600 nm (OD_600_) of each culture was determined, and the culture concentration was adjusted to have 1 × 10^7^ bacteria per ml in LB by converting the OD to an estimated number of CFU per milliliter using a standard curve. For biofilm formation on protein-coated surfaces, ELISA plates (catalog no. DY990; R&D Systems, Minneapolis, MN, USA) were coated overnight at room temperature with 10 μg/ml mouse laminin (catalog no. 23017015; Thermo Fisher Scientific, Waltham, MA, USA) or 1% BSA in phosphate-buffered saline (PBS). The plates were subsequently blocked with 1% BSA, followed by fluid aspiration and air drying before concentration-adjusted P. aeruginosa cultures were added. Adjusted cultures were grown at 37°C for 18 h. The plates were subsequently washed and stained with 0.1% crystal violet (CV). The plates were washed to remove excess dye and air dried before the addition of 30% acetic acid to solubilize the CV. The solubilized CV was quantified in a plate reader at 550 nm.

### Growth kinetics of P. aeruginosa strains.

Overnight LB P. aeruginosa cultures were centrifuged, washed, and resuspended in fresh LB before the OD_600_ for cell concentration adjustment was measured. Bacteria were seeded at 1 × 10^6^ cells per 100 μl LB in a transparent 96-well plate. The plate was covered with a lid and incubated in a plate reader at 37°C for 24 h. The reader was programmed to measure the OD_600_ every 10 min after shaking the plate for 5 s.
